# Crypsis by background matching and disruptive coloration as drivers of substrate occupation in sympatric Amazonian bark praying mantises

**DOI:** 10.1038/s41598-023-46204-x

**Published:** 2023-11-15

**Authors:** João Vitor de Alcantara Viana, Rafael Campos Duarte, Camila Vieira, Pablo Augusto Poleto Antiqueira, Andressa Bach, Gabriel de Mello, Lorhaine Silva, Camila Rabelo Oliveira Leal, Gustavo Quevedo Romero

**Affiliations:** 1 Programa de Pós-Graduação em Ecologia, Instituto de Biologia, Universidade Estadual de Campinas, Campinas, São Paulo Brazil; 2 Laboratório de Interações Multitróficas e Biodiversidade, Departamento de Biologia Animal, Instituto de Biologia, Universidade Estadual de Campinas (UNICAMP), CP 6109, Campinas, São Paulo CEP 13083-970 Brazil; 3 Universidade Federal Do ABC, São Bernardo Do Campo, São Paulo CEP 09606-045 Brazil; 4 Centre for Ecology and Conservation, College of Life and Environmental Sciences, University of Exeter, Penryn Campus, Penryn, TR10 9FE UK; 5 Departamento de Ciências Básicas, Universidade de São Paulo (USP), Campus de Pirassununga, Pirassununga, São Paulo CEP 13635-900 Brazil; 6 Programa de Pós-Graduação Em Ecologia E Conservação da Biodiversidade, Instituto de Biociências, Universidade Federal de Mato Grosso, Avenida Fernando Corrêa da Costa, N° 2367, Boa Esperança, Cuiabá 78060900 Brazil

**Keywords:** Ecology, Behavioural ecology

## Abstract

Background matching and disruptive coloration are common camouflage strategies in nature, but few studies have accurately measured their protective value in living organisms. Amazon's Bark praying mantises exhibit colour patterns matching whitish and greenish-brown tree trunks. We tested the functional significance of background matching and disruptive coloration of different praying mantis morphospecies (white, grey and green) detected by DNA barcoding. Through image analysis, avian visual models and field experiments using humans as potential predators, we explored whether the background occupation of mantises provides camouflage against predation. Data were obtained for individuals against their occupied tree trunks (whitish or greenish-brown) and microhabitats (lichen or bryophyte patches), compared to non-occupied trunks. White and grey mantises showed lower colour contrasts against occupied trunks at the scale of tree trunk, with no differences in luminance contrasts. Conversely, green mantises showed lower colour and luminance contrasts against microhabitats and also exhibited high edge disruption against greenish-brown trunks. The camouflage of white and green mantis models against colour-matching trunks increased search time and reduced encounter distance of human predators. We highlight the importance of camouflage strategies at different spatial scales to enhance individual survival against predators. Specifically, we present a stunning study system to investigate the relationship of phylogenetically related species that use camouflage in sympatry.

## Introduction

Two core determinants of the life history of predators that are not at the top of food chains are not being eaten and holding a suitable habitat for ambush and hunting their prey^1^. This “survival equation” of life is determined by making-decision for rentable hunting sites that contain available prey, lower predation risks and reduced competitive rates, as well as for backgrounds with proper physical conditions^1,2^. The use of habitats to catch prey and reduce predation risks can be mediated by the selection of microhabitats, that is, small or limited portions of habitat that differs in the appearance from the surrounding broad habitat, with distinctive ecological traits more suitable for a given species^3^.

One of the most widespread protective strategies used among prey and predators is visual camouflage^4^. These adaptations to become undetected or unrecognized have intrigued researchers since the observations made by pioneer naturalists such as Wallace (1867)^5^ as well as crossed borders for the arts, in which Thayer (1909)^6^ sets out the initial evolutionary hypothesis for camouflage. Successful camouflage combines multiple ecological conditions, such as organism coloration, ambient lightness, the sensory-cognitive capability of the observer, background contrast, and animal behaviour^7,8^. Camouflage includes several strategies that act in different routes in the sensory and cognitive systems and prevent detection or recognition by the viewer^9^. Among these strategies, background matching and disruptive coloration are widespread in nature and can act simultaneously in the same organism^10–12^. Background matching occurs when body colour patterns generally match the colour, lightness, and pattern of the background, reducing the feature information between the appearance of an organism and its general or specific surroundings^11^. Contrary, disruptive coloration is defined by the presence of highly contrasting coloration patterns that blur the outline and break up the real surface form of the organism, impairing the detection or recognition of real body configuration in the sensory system of the viewer^9,13^.

Background choice is a widespread behaviour in nature that operates at species, individual or morph levels^8^. It is considered a key factor in the selection of suitable habitats and microhabitats by providing prey camouflage and increase individual survival^14,15^. Classical theoretical predictions about optimal cryptic coloration in heterogeneous habitats suggest trade-offs between two visual backgrounds^15^. The predicted optimal coloration is a function of the degree of prey crypsis in each background and its probability of occurring in the microhabitats, as well as the probability of encountering a predator at those sites. Such relationship can be optimized by behavioural selections^15^. Background choices can benefit prey through a better matching with a generalized (compromise coloration) or specific portion of backgrounds (microhabitat), as well as increase prey survival through choices for visual complexity backgrounds that override or act together with background matching^16^.

Amazon rainforests hold countless habitats that invertebrate predators can use as sites for both concealment and prey capture. Bark mantises (Liturgusidae) comprise a group of praying mantises strictly associated with tree bark habitats^17^. They have specialized morphological adaptations such as dorsoventrally body flattening for a lower profile against tree trunks and several patterns of cryptic coloration (e.g., background matching and disruptive coloration)^18^. In Neotropical regions, two major Liturgusidae tribes are present, namely Liturgusini, with four genera (*Corticomantis*, *Fuga*, *Liturgusa*, and *Velox*), and Hagiomantini, with one genus (*Hagiomantis*)^17,19^. Both tribes are highly dependent on camouflage as an anti-predatory strategy, with individuals showing preferences to occupy smooth trunks that favour running. Although bark praying mantises have wings, these structures are rarely used to escape from predators, except in situations where individuals are very disturbed and fly to another tree^16^.

Apart from the similarities in the pattern of trunk occupation, there is a significant lack of knowledge regarding the behaviour and life history of the praying  mantises of these Liturgusidae tribes. We observed Liturgusidae mantises on lowland Amazonian rainforest occupying both whitish tree trunks covered by random patches of lichen, and brown tree trunks covered by random patches of green bryophytes (i.e. backgrounds). We recorded white praying mantises exhibiting colour patterns that resembled lichen-covered tree trunks where they were exclusively found. Oppositely, we also recorded green mantises occurring in greenish-brown trees covered by random patches of bryophytes. In addition, we also found grey individuals occupying a reforested area composed of whitish trees also covered by random patches of lichens.

Based on these assumptions, we aimed to investigate if the background occupation by Liturgusidae Amazonian bark praying mantises is related to camouflage benefits against potential avian predators at different backgrounds and spatial scales. Backgrounds are defined as whitish and greenish-brown tree trunks. Spatial scales are defined as tree trunk, i.e., a broad selection of the trunk substrate, and microhabitats, i.e., trunk areas covered with lichen and bryophyte patches. Neotropical praying mantises are poorly studied and difficult to identify as juveniles, therefore, we first used DNA barcode analysis to test whether the different Liturgusidae colour morphs observed in the field corresponded to distinct species, from which we detected three morphospecies associated to white, grey and green body colour patterns. Further, we used image analysis and visual modelling to test the effectiveness of background matching and disruptive coloration as possible camouflage strategies employed by bark mantises to avoid predation. To test background matching, we evaluated colour and luminance contrasts of individuals of each morphospecies to their occupied trunks (i.e., comparing praying mantises against a broad selection of whitish trunks, for the white and grey morphospecies, and greenish-brown trunks, for the green morphospecies, observed in the study area) and microhabitat (i.e., comparing praying mantises against lichen patches for the white and grey morphospecies or against the bryophyte patches for the green morphospecies). We also calculated praying mantises colour and luminance contrasts against the non-occupied background at the tree trunk scale (i.e., white and grey mantises against greenish-brown tree trunks and green mantises against whitish trunks). In order to assess disruptive coloration, we used Gabor filters—GabRat analysis^20^ to test the salient and coherent edges of praying mantises against their own background. We also tested edge disruption (GabRat) of praying mantises against non-occupied backgrounds at the scale of tree trunk (as above).

Both types of tree trunks exhibit varying levels of visual background heterogeneity. Whitish trees have primarily a white colour but exhibit variations in brightness due to the presence of random lichen patches. In contrast, greenish-brown trees exhibit a high colour pattern heterogeneity due to the covering of bryophyte patches over their brown-coloured trunks. Based on that, we predict that individuals of the white and grey morphospecies will exhibit lower colour and luminance contrasts as well higher GabRat values on whitish trunks, while individuals of the green morphospecies will show lower colour and luminance contrasts as well as higher GabRat values against bryophyte-covered tree trunks.

Finally, we aimed to test the effectiveness of camouflage by background matching employed by the individuals of the white and green morphospecies against their occupied trunks to reduce the detection of potential predators. Currently, common approaches testing similar questions use human participants as a proxy of natural predators in citizen science online games and “predation” experiments in the field^21^. Recent research has revealed no significant differences between visual processing of searching behaviour between humans and birds, despite their differences in cognitive and sensory capabilities^21–23^. Therefore, we performed a field experiment with humans searching for white and green paper models of praying mantises against greenish-brown and whitish tree trunks. Our hypothesis is that prey models with similar coloration to the background (e.g., white models on whitish lichen-covered tree trunks) will lead to predators taking longer time and needed to stay at shorter distances from the subject to recognize and identify them compared to models with more contrasting coloration to backgrounds (e.g., white models on bryophyte covered greenish-brown tree trunks).

## Material and methods

### Study site and sampling of praying mantises

The study was conducted in December 2021 and October 2022 in an area of the Amazon rainforest at São Nicolau Farm, located in the southern Amazonian biome and northwest of Mato Grosso state, Brazil (09° 51' 16″ S; 58° 14' 57″ W). The São Nicolau Farm holds 7000 ha of open and dense rainforest and 2000 ha of reforested forest and cattle pasture^24^. We extensively searched for praying mantises through systematic visual scans of trunks between 0 to 3 meters high on whitish (lichen-covered) and greenish-brown (bryophyte-covered) tree trunks in natural and reforested areas of the farm. Photographs for objective measurement of praying mantises coloration are not feasible without collections (Authors, *personal observations*). Thus, we captured each individual with plastic pots and bags by cautiously holding them against the trunks before the capture. We carefully transported the individuals to the field laboratory and placed them in a freezer for 3–5 min to reduce their metabolism in order to obtain photos without any movement (see details below). After photography, praying mantises were euthanized and kept in absolute alcohol for molecular analyses.

**Figure 1:**
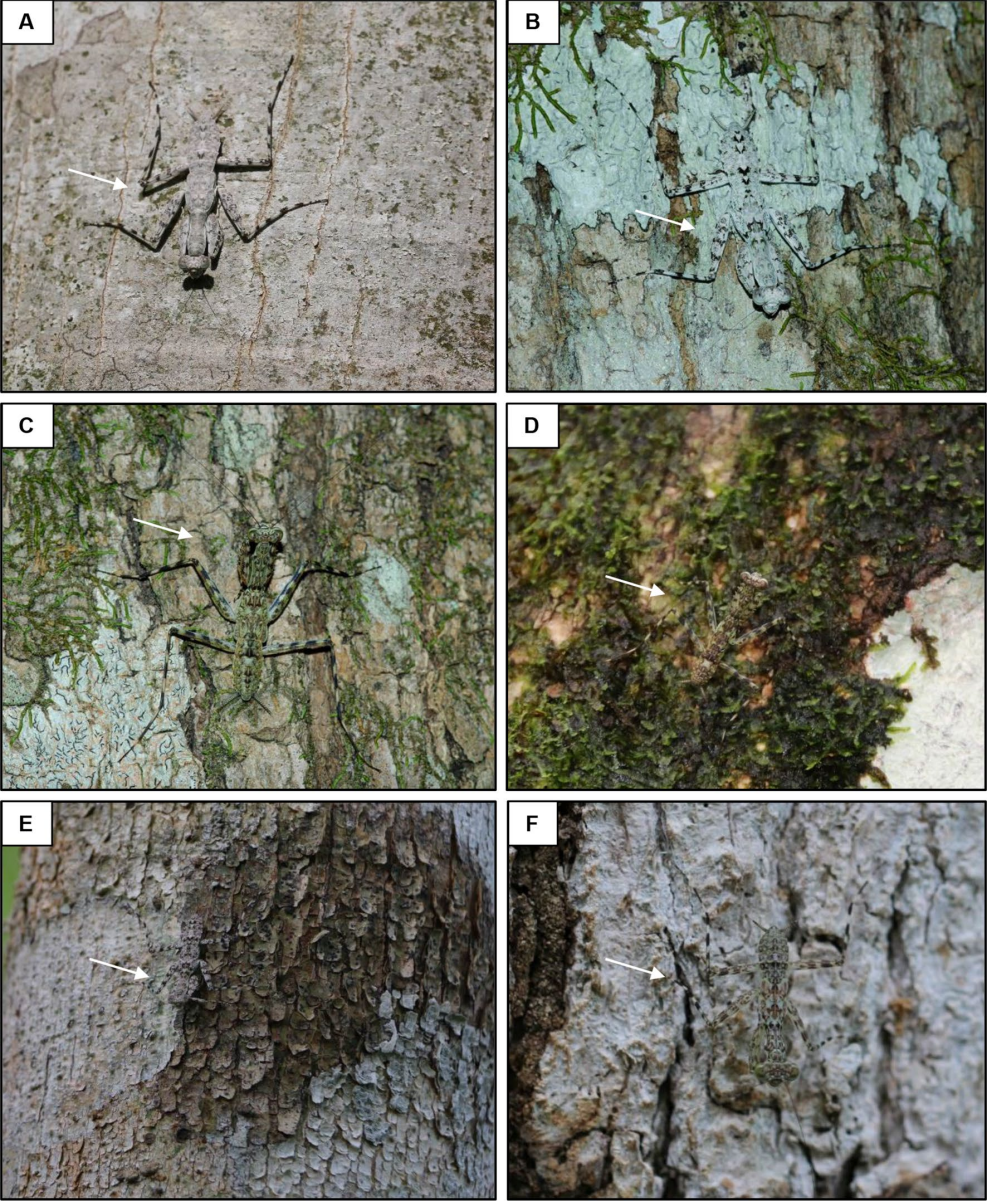
Background occupation of different praying mantis morphospecies in tree trunks of the southern Amazon rainforest. Two individuals of the white morphospecies (*Hagiomantis* sp.) resting on (**A**) a whitish trunk (tree trunk scale) and (**B**) a patch of lichen covering a whitish tree trunk (microhabitat scale). Two individuals of the green morphospecies (*Liturgusa* sp.) resting on (**C**) a greenish-brown trunk (tree trunk scale) and (**D**) a patch of bryophyte covering a greenish-brown tree trunk (microhabitat scale). Two individuals of the grey morphospecies (Liturgusidae) resting on (**E**) a whitish trunk (tree trunk scale) and (**F**) a patch of lichen covering a whitish trunk (microhabitat scale).

We collected juvenile individuals of white (*n* = 12), green (*n* = 10) and grey (*n* = 5) colour types. White individuals occurred only in border edges of the forest and reforested areas, resting on whitish trunks of *Ficus maxima*, *Hymenaea* spp., *Croton* sp., *Anadenanthera colubrine,* and *Tabebuia* sp., all covered with random patches of lichens. Green individuals were found in open and dense forest formations resting on greenish-brown tree trunks of *Acacia* sp., *Handroanthus albus* and *Senegalia polyphylla*, all covered with random patches of bryophytes. Grey mantises were found in whitish tree trunks of *Croton* sp. and *Tabebuia* sp. also covered with random patches of lichens in a specific reforested location (Fig. 1).

### Molecular identification of bark mantises morphotypes

We previously used a dichotomous key for genus of Mantodea to separate morphotypes at the lowest possible taxonomic level^17^. In order to correlate morphological patterns and the genetic identity of individuals, we evaluated the barcode region of the mitochondrial encoded cytochrome c oxidase I (COI) gene^25^. The use of molecular identification allows us to evaluate if the colour pattern of individuals results from intraspecific variation or genetic differentiation between separate species.

Total genomic DNA (white morphospecies: *n* = 7, grey morphospecies: *n* = 2, green morphospecies: *n* = 4, Table S1) was extracted according to the standard procedure of the DNeasy Blood and Tissue extraction kit (Qiagen, Hilden, Germany), then stored at -20 °C. DNA concentration and quality were estimated with a UV NanoDrop spectrophotometer (ThermoFisher Scientific, Waltham, MA, USA). The mitochondrial gene cytochrome c oxidase subunit I (COI, 5′ end, ca. 640 bp) was amplified using the primers LCO-F^26^ + Nancy-R^27^. The conditions for amplification were: 2 μL of genomic DNA; 2.5 μL of 5 × buffer; 2.5 μL of 5% DMSO; 2 μL of MgCl2 at 25 nM; 0.4 μL of dNTP at 10 nM; 0.5 μL of each direct and reverse primer at 10 nM; 0.2 μL de Taq DNA Polymerase (Promega, Madison, WI, USA); and autoclaved deionized H_2_O in sufficient quantity for 26 μL of reaction. The PCR program was set up as follow: initial denaturation at 94 °C (2 min); 34 cycles of denaturation at 94 °C (45 s); annealing at Ta°C (45 s; Table S1) and extension at 72 °C (1 min); a final extension cycle at 72 °C (7 min).

All amplified samples were run on a 1% agarose gel with 50 mM Tris–acetate (TAE) buffer (pH 7.5–7.8) to test the quality of amplification before sequencing procedures. The resulting DNA fragments were purified with the ExoSAP-IT (GE Healthcare, Bucks, UK) and sequenced with the primers used for amplifications in an ABI 3500 × L automated sequencer (Life Technologies) with Big Dye Terminator v.3.1 kit (Applied Biosystems). Sequences were edited using the software Chromas v.2.6.6 (Technelysium Pty Ltd) and compared to GenBank database^28^ through BLAST^29^ to confirm their identity as COI gene of Liturgusidae.

Sequences of 640 bp were aligned with MUSCLE^30^ executed in Mega v11.0.13^31^ and deposited in GenBank (Table S1). Pairwise genetic distances between individuals were estimated assuming Kimura two-parameter as nucleotide substitution model^32^ and used to reconstruct a neighbour-joining tree in Mega v7.0.26, using as outgroup the praying mantis *Theopompella chopardi* (Liturgusidae) (GenBank accession number: EF383918.1). Branch supports were estimated by 10,000 bootstrap replicates.

### Digital photography and image analysis

#### Photography

Photography of animals and backgrounds followed standard protocols^33^. Digital photographs were taken with a Nikon D7000 camera converted to full-spectrum sensitivity by removing the UV and IR blocking filter to enable UV sensitivity and fitted with a 105-mm Micro-Nikkor lens. Human visible photographs were taken by using an ultraviolet-infrared Kolari Vision UV-IR Cut filter, allowing the capture of only visible light spectrum (from ~ 400 to 700 nm), and UV photographs were taken with a UV pass filter (Optic Makario) allowing the capture of only ultraviolet light (from ~ 300 to 400 nm). Changes in ambient lighting conditions were controlled by photographing one well-homogenized pellet of barium sulphate (reflecting 99% of light) placed in each image.

Photographs of praying mantises and tree trunks (greenish-brown trunks = 12; whitish trunks = 17) were taken with a tripod outside of the laboratory and in the field, respectively, under natural illumination and using light diffusers and contained a scale bar in the same plane as the subjects. Trunk photographs were taken at 1 m from the trees and 1 m height in the North positions. All photos were taken on sunny days with a fixed aperture of F8 (ISO 400) and saved as raw images^33^.

We used the ‘Multispectral Image Calibration and Analysis (MICA)’ toolbox, an Image-J plugin for creating and calibrating multispectral images as well as to run all the subsequent image analyses^33^. Visible and UV photos were first aligned, with the white standards being used to equalize the pixel responses to lighting conditions, which resulted in multispectral images.

After calibration, we marked regions of interest (ROIs) on images of both praying mantises and their occupied backgrounds for colour measurements (see details below)^33^. For this purpose, we marked ROIs on the dorsal surface of each praying mantis individual, excluding the appendices, and compared them with the ROIs of the backgrounds. For the backgrounds, we marked ROIs for each tree trunk within two scales, one for tree trunk comparisons, as the broad area of the trunk (i.e., whole trunk; ROI defined as a square with a side of 10 cm) and another for the specific microhabitat portion (i.e., lichen and bryophyte patches; ROI defined as a square with a side of 2 cm). For the tree trunk scale, we consistently used ROIs at the same central position of each tree trunk photo, even when it exhibited some degree of background heterogeneity (e.g., patches of bryophytes and lichens). However, for the microhabitat scale, we specifically selected one lichen or one bryophyte patch per tree trunk.

#### Background matching analysis

We used the Receptor Noise-Limited Model (RNL) to quantify the colour and luminance matching between praying mantises and both occupied and non-occupied backgrounds^34^ Here we defined 'occupied backgrounds' as the specific type of tree trunk (either greenish-brown or whitish) on which each mantis morphospecies was found. The occupied trunks also closely correspond to the body coloration of the respective mantis morphospecies. Model calculations resulted in just noticeable differences (JNDs), which is a metric of colour/luminance discriminability between two objects by a potential viewer. Values below 1.00 indicate that the viewer is unable to discriminate the two objects, with the object detectability increasing as JND values increase^34,35^. Since passerine birds are common arthropod predators and exhibit a conservative visual system across different species^35^, we used the visual model of the blue tit (*Cyanistes caeruleus*) in our analysis. Blue tits are UV-sensitive birds and have been extensively used as models in several studies about arthropod coloration and camouflage^21,36, 37^. We preferred to use the D65 irradiance spectrum as a measure of incident illumination in our model^38^ since although the sampling of praying mantises occurred in both forest and reforested areas, these landscapes are open habitats and are not considered as closed and shaded as typical Amazon forest areas.

After modelling, we calculated achromatic (= luminance, based on double cones responses) and chromatic (= colour) contrasts of each praying mantis morphospecies against tree trunks. We considered both the tree trunk and microhabitat spatial scales in the occupied background as well as the tree trunk scale of the non-occupied backgrounds (e.g., white and grey morphospecies *vs.* greenish-brown trunks and green morphospecies *vs.* whitish trunks).

#### Disruptive coloration analysis

In order to understand how disruptive the praying mantis morphospecies coloration was against the different trunk types, we estimated on each image the false and coherent edges of the dorsal praying mantis' surfaces through the GabRat tool in the MICA toolbox^20,33^. This tool is based on the Gabor band pass filter, an angle-sensitive filter, which has an algorithm that measures the ratio of the true outline edges of mantises compared to false edges^20^. We also used blue tit visual model, reducing our channels to the double cones, and set an acuity value of 6 cycles per degree at 1 m distance because these values closely resemble the acuity for other small avian predators that forage on trunks^20,39,41,42^. We followed previous recommendation and set the sigma value of Gabor filters as 3 units^20^. The GabRat values were calculated by randomly placing each mantis ROI against each trunk image of both occupied and non-occupied substrates over 10 different positions, without overlap, with the values being averaged subsequently to generate a single value per individual. This procedure resulted in a total of 24, 10 and 20 averaged GabRat values for the white, grey and green morphospecies, respectively. Higher rates of false edges to coherent edges (i.e., higher GabRat) indicate increasing disruption of body edges and consequently greater difficulty in target detection, while lower values suggest salient coherent edges and ease of viewer detection. GabRat ranges between 0 and 1, with values below 0.20 indicating low disruption, between 0.20 and 0.40 intermediate, and above 0.40 considered highly disruptive^20,41^.

### Field predation experiments

We carried out a field predation experiment using paper praying mantis models to understand the camouflage benefits of the different Liturgusidae morphospecies on lichen and bryophyte-covered trees. For this, we created models of the white (*Hagiomantis* sp.) and green (*Liturgusa* sp.) morphospecies, as those are the most abundant and contrasting colour types in the study area. The artificial models were designed to represent the real body colour patterns of white and green bark mantises as similar as possible, except by the exclusion of their legs (Supplementary Material 1). For that, we calibrated the multispectral images of white (*n* = 10) and green (*n* = 10) praying mantises to human vision (following the same protocol to convert to bird vision) and used them to create models whose shape resembled the real silhouette and body spot patterns of each morphospecies using Adobe Photoshop (version 2.2.0). To obtain the most accurate colour for each model, we first created and printed a set of filled squares with candidate colorations for white and green models. The printed colour patches were then photographed and their RGB values were measured and compared to the reflectance values of real mantises, modelled for human vision, with the most similar values being selected for both green and white models (see Supplementary data for additional information).

Models were printed with a laser Konica Minolta—Bizhub C364 printer on waterproof photo paper and exhibited comparable size with real bark mantises (0.5 cm in width and 3.2 cm in length). In the field, models were fixed to tree trunks using thumbtacks, which were glued to the back of models using high-resistance and quick-drying instant glue (Superbonder®). Models were inserted in pairs (one green and one white model) into each tree trunk in random positions regardless of microhabitat type (lichen or bryophyte patches), varying from 80 to 140 cm height, making them visible to all human predators. The trunks where models were placed belonged to two whitish (*Anadenanthera colubrina* and *Genipa americana*) and three greenish-brown tree species (*Acacia* sp., *Handroanthus albus* and *Senegalia polyphylla*), which were spaced at least 10 m apart. Trees were carefully selected so that the models in subsequent trees could not be viewed at the same time by the participants.

The participants (*n* = 21) were guided through a path of pre-selected trees (*n* = 6) that randomly varied in the colour of their trunks between whitish and greenish-brown. The selected trees and the initial distance of the path (8 m) were marked to standardize the sampling effort of each participant. Before starting, all participants were instructed about the procedures of the experiment and how the models were fixed on the trees. We quantified the searching time (ST) taken by each participant to recognize each praying mantis model as well as the encounter distance (ED) travelled to find models. To identify possible misunderstanding and guessing on the identification of models by the participant, one experienced researcher (AB, LS and GM) was positioned next to the tree to check if the target was identified. As soon as the first model was found by the participant, the researcher paused the time and recorded both the ST and ED for the first target. After that, the researcher warned the participant and restarted the time, so that the subject could approach and find the next model at the same trunk. After finding the two targets, the participant continued to the other trees until completing all the experiment path (approximate distance covered of 80 m between the trees). The gender and age of each participant were recorded. Additional information about the experimental protocol can be found in Supplementary data and Supplementary material (Figure S1).

### Ethics statement

Fieldwork was conducted under the permission of ONF Brazil-São Nicolau Farm. Sampling collections follow the environmental Brazilian rules and were granted by the SISBIO (73795-1). The Comitê de Ética em Pesquisa (CEP)—Human Ethics Committee granted ethical approval for the human predation experiment (CEP—65017422.5.0000.5404).

### Statistical analyses

All statistical analyses were undertaken using the software R v.4.2.2^43^. We used separated linear mixed-effects models to test “colour” and “luminance” contrasts of each colour type between greenish-brown and whitish trunks as well as against lichen patches (for white and grey mantis) or bryophyte patches (for green mantis). For all models, the JND values were treated as the response variable and the trunk backgrounds as the fixed factor, with the praying mantis identity set as a random factor to control for repetitive measurements made on the same individual. In this way, each praying mantis morphospecies was compared against occupied tree trunks, microhabitats within occupied trunks, and non-occupied tree trunks^44^. The same model structure was used to compare the GabRat values of the praying mantis colour types between trunks, but now considering only greenish-brown and whitish trunks. We also used linear mixed-effects models to test the effectiveness of camouflage of white and green praying mantises artificial models on greenish-brown and whitish trunks in the field predation experiment. Mixed-models were fitted separately for the two response variables (searching time—ST, encounter distance—ED), with the background (greenish-brown and whitish trunks) and the mantis model (green and white) set as fixed factors, the participant (= predator) identity as a random factor and the trunk identity as a random factor nested within background to control for data dependence, since two models of different treatments were always placed paired on the same tree. All models were fitted through the *lmer* function of the *lme4* package^45^ and the associated significance tests through the *anova* function of the *lmerTest* package^46^. Model residuals were checked visually for normal error distribution using q-q plots, and the homogeneity of variances was tested using the Levene test in R, for which the colour and luminance JNDs as well as the Gabrat values for all praying mantis colour types, except the luminance contrast of the white morph and the GabRat of the grey morph, required log transformation to meet model assumptions. Similarly, the ST data of the field predation experiment also required log transformation. Finally, in the case of significant effects, we performed Tukey post hoc tests to assess differences between factor levels using the *emmeans* function of the *emmeans* package^47^.

## Results

### Molecular analysis: DNA barcoding

**Figure 2:**
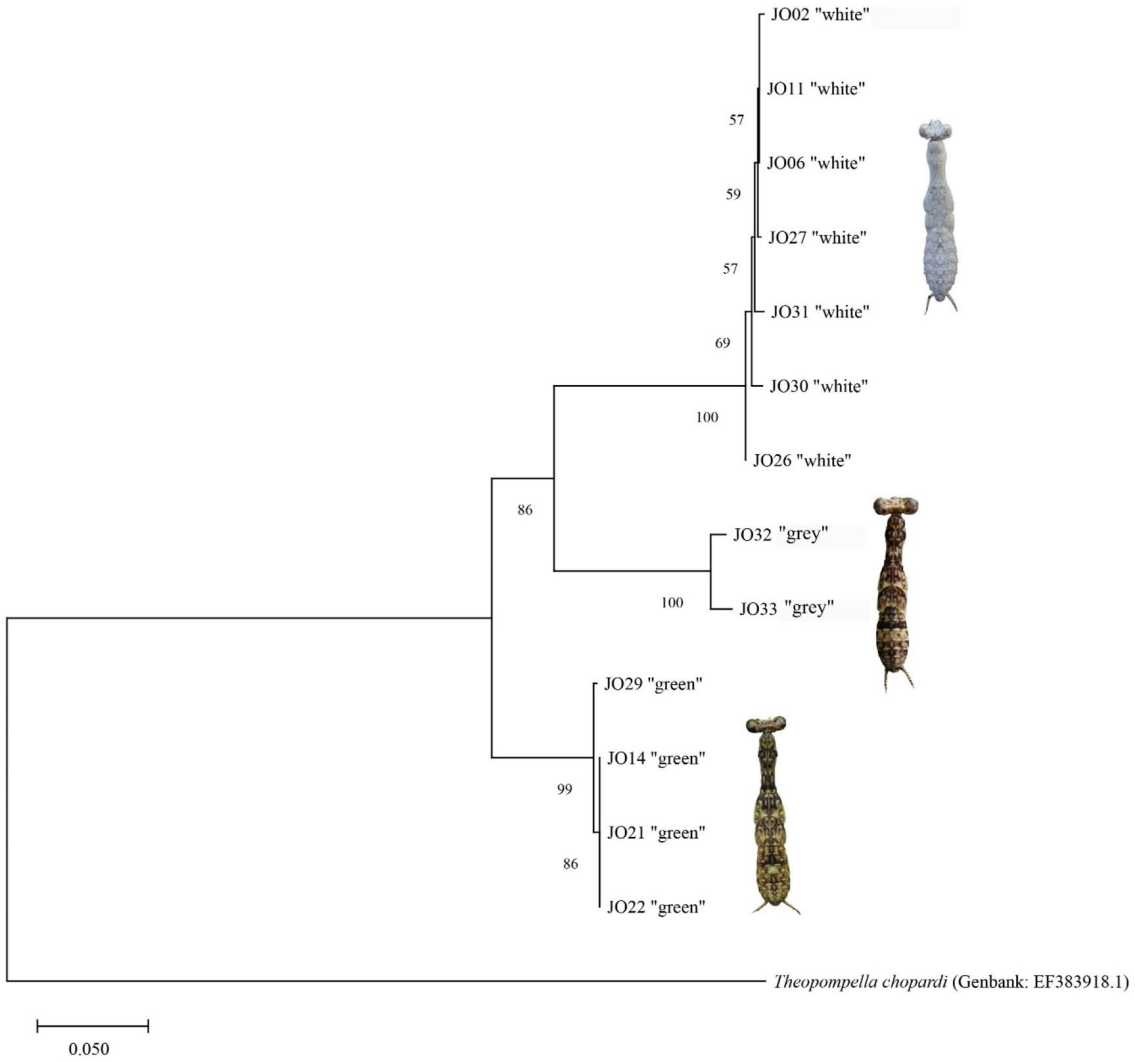
Phylogenetic tree estimated by neighbour joining and built considering Kimura two-parameter distance between praying mantis specimens and using *Theopompella chopardi* as outgroup (GenBank accession number: EF383918.1). Branch support based on 10,000 bootstrap replicates is shown.

The DNA barcode analysis evidenced that the white, grey and green bark mantis colour types are not only different species but belong to different genera (Table S2, Fig. 2). After searching for similar COI sequences in the GenBank database, we identified that the white colour type belongs to the *Hagiomantis* (Serville, 1839) genus while the green morphotype to the *Liturgusa* (Saussure, 1869) genus. However, there were no matching sequences for the grey morphospecies (Liturgusidae), indicating that either the sequences for this species have not been included in the GenBank database yet or it represents a previously undescribed group of praying mantises. From this point forward, we considered the grey praying mantis as an unidentified species of the Liturgusidae family and performed all comparisons independently for the three morphospecies considering them as separate colour types.

### Background matching

**Figure 3:**
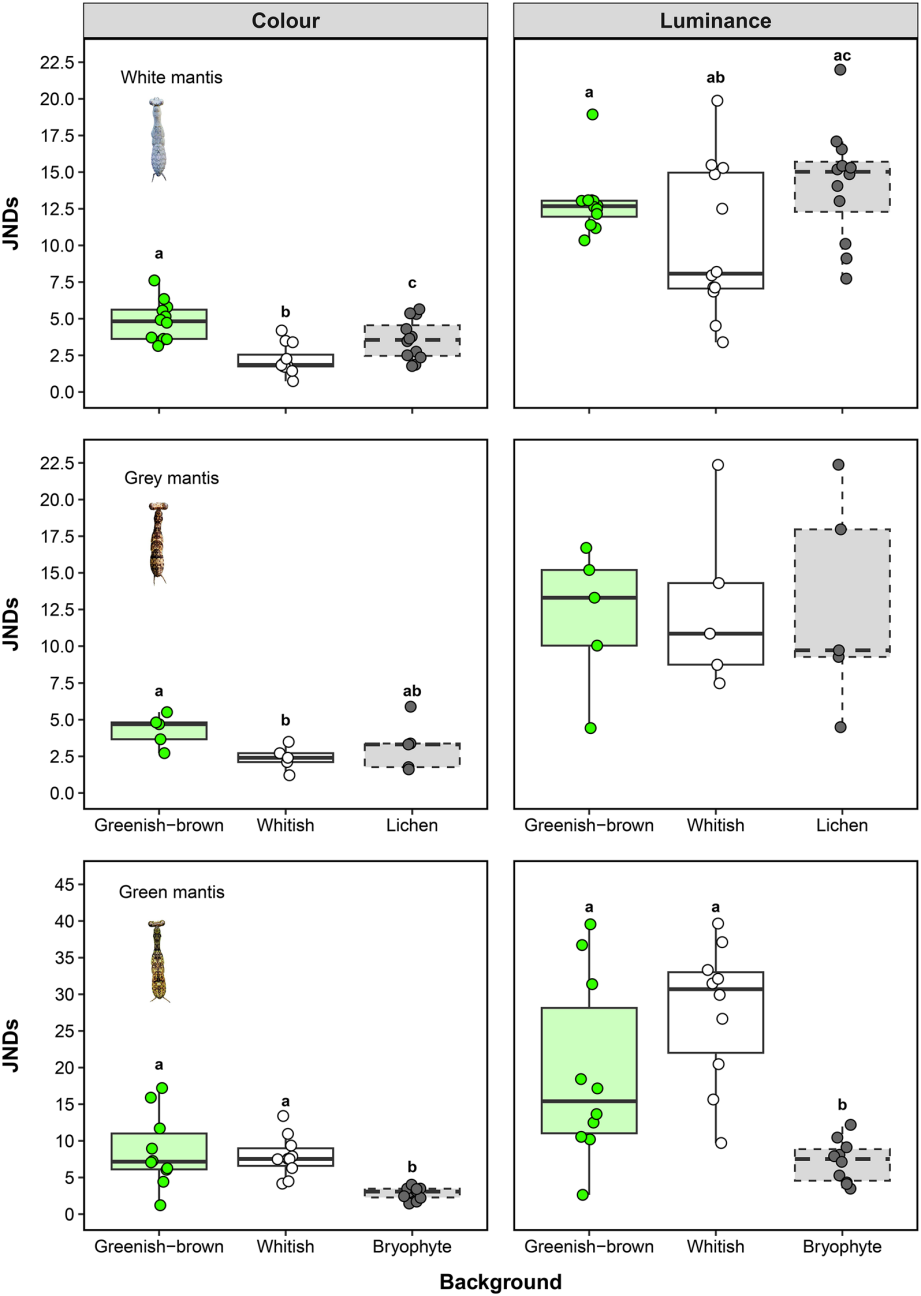
Colour and luminance contrasts of white (*Hagiomantis* sp.), grey (Liturgusidae) and green (*Liturgusa* sp.) praying mantises against different trunk backgrounds in the southern Amazon rainforest. Contrasts are expressed as JND (just-noticeable-differences) units based on the vision of the blue tit (*Cyanistes caeruleus*), in which lower values indicate better matching. Here and in the next figure, boxes display medians and interquartile ranges (IQRs), whiskers represent the lowest and highest values within 1.5 × IQRs and circles represent raw data, on which a random noise was added to avoid overlap. Boxes with solid contour lines refer to trunk backgrounds (e.g., greenish-brown and whitish trunks) while boxes with dashed contour lines refer to microhabitat backgrounds (e.g., patches of lichen covering whitish trunks for white and grey mantis, and patches of bryophytes covering greenish-brown trunks for green mantis). Different letters indicate significant differences between background types (*p* < 0.05).

With the exception of the achromatic contrasts for the grey praying mantises, the colour and luminance contrasts of the other mantis morphospecies differed between trunk backgrounds (Table 1) but the scale at which individual camouflage was optimized depended on the mantis species. The individuals of the white morphospecies exhibited lower colour contrasts against whitish trunks (mean ± se: 2.20 ± 0.28), but their luminance contrasts did not vary considerably between backgrounds (Fig. 3). A similar pattern was observed for the individuals of the grey morphospecies, which exhibited lower colour JNDs against whitish trunks (2.39 ± 0.37) in comparison to the other backgrounds but no differences regarding luminance contrasts. In opposition, individuals of the green morphospecies showed better chromatic and achromatic matching within the microhabitat scale, exhibiting lower colour (2.84 ± 0.27) and luminance (7.21 ± 0.92) JNDs against bryophyte patches in comparison to whole greenish-brown or whitish trunks (Fig. 3).

**Table 1:** Background matching and disruptive coloration of praying mantis of different morphospecies against trunk backgrounds in the southern Amazon rainforest. Summary results of the analysis of variance applied to linear mixed-effects models testing differences in colour and luminance contrasts (as JNDs—just-noticeable differences) and edge disruption (as GabRat) based on the vision of the blue tit (*Cyanistes caeruleus*) between different trunk backgrounds. For all models, mantis identity was included as a random factor to control for repeated measurements made in the same individual. Significant differences are shown in bold.

		Colour JNDs			Luminance JNDs				GabRat		
	*df*	*MS*	*F*	*p*	*MS*	*F*	*p*	*df*	*MS*	*F*	*p*
White mantis											
Background	2	2.130	25.54	** < 0.001**	47.993	3.55	**0.046**	1	0.029	0.97	0.347
Residuals	22	0.083			13.522			11	0.030		
Grey mantis											
Background	2	0.477	4.82	**0.042**	0.010	0.03	0.967	1	2.27e^-6^	0.001	0.98
Residuals	8	0.099			0.291			4	0.002		
Green mantis											
Background	2	3.233	18.36	** < 0.001**	4.619	17.34	** < 0.001**	1	0.847	9.28	**0.014**
Residuals	18	0.176			0.266			9	0.091

### Disruptive coloration

**Figure 4:**
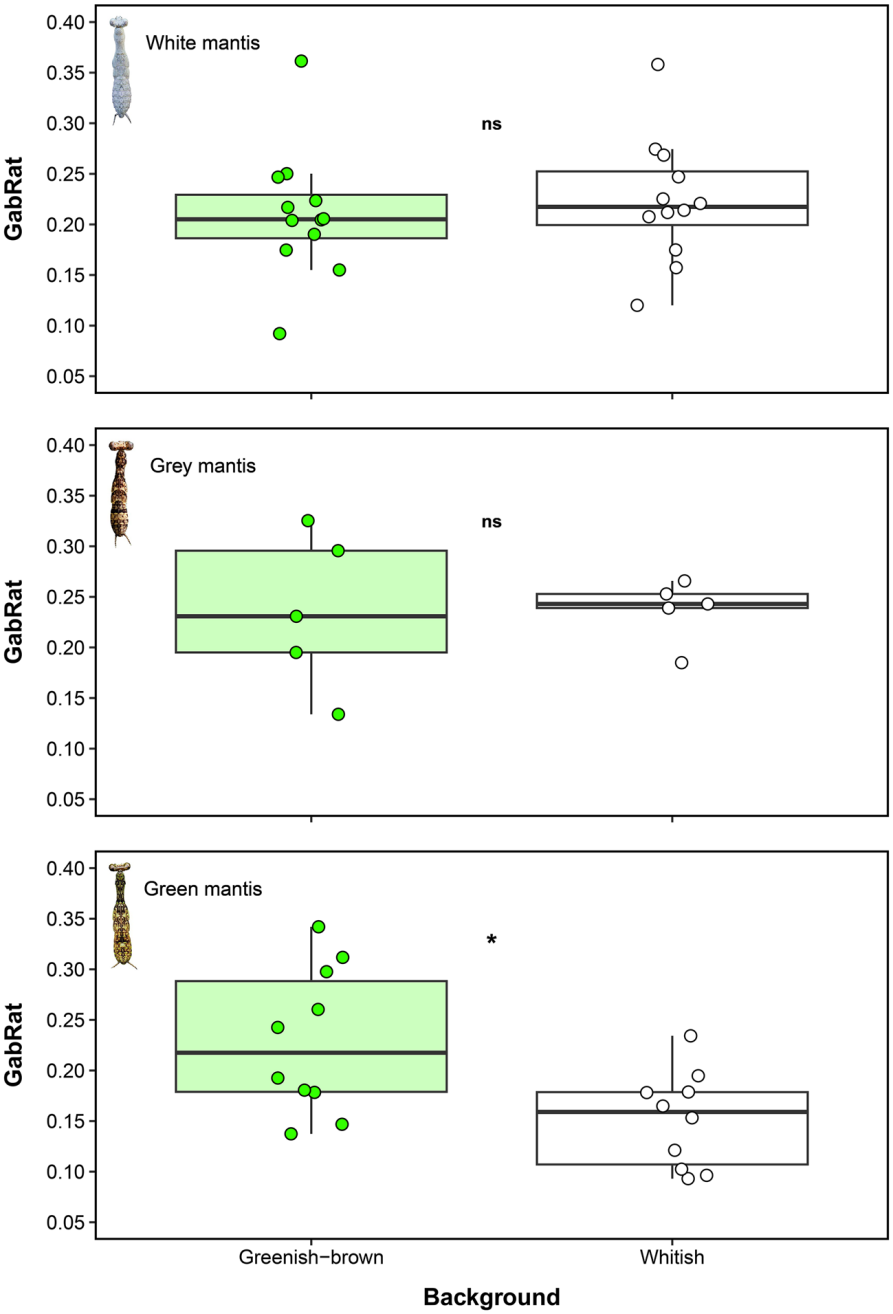
Edge disruption of white (*Hagiomantis* sp.), grey (Liturgusidae) and green (*Liturgusa* sp.) praying mantises against different trunk backgrounds in the southern Amazon rainforest. Edge disruption is expressed as GabRat, which is a metric comparing the ratio between false to coherent edges based on the vision of the blue tit (*Cyanistes caeruleus*), in which larger values indicate higher disruption. The asterisk (*) denotes significant differences between factor levels (*p* < 0.05), whereas *ns* indicates non-significant differences.

Regardless of the background, individuals of the three praying mantis morphospecies exhibited intermediate levels of edge disruption (GabRat values between 0.20 and 0.40) when viewed by a potential avian predator (Fig. 4). There was no difference in the mean GabRat of both white (*Hagiomantis* sp.) and grey mantis (Liturgusidae) between greenish-brown and whitish trunks. However, a significant effect was observed for green mantis (*Liturgusa* sp.), indicating that greenish-brown trunks promote higher levels of edge disruption when compared to whitish trunks for the individuals of this morphospecies (Table 1, Fig. 4).

### Field predation experiment

**Figure 5:**
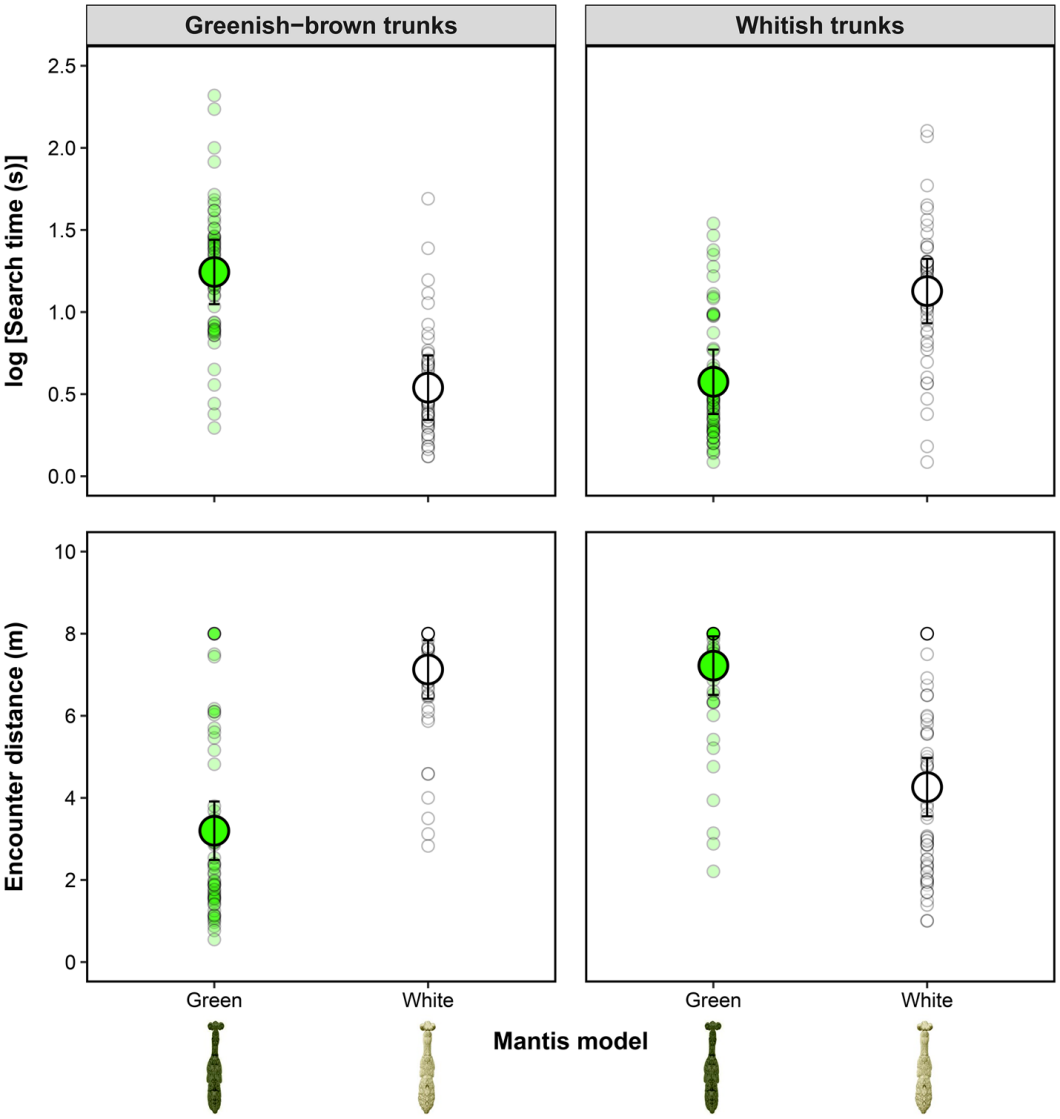
Searching time (log-transformed, in seconds) and encounter distance (in meters) of human predators “hunting” paper models resembling green (*Liturgusa* sp.) and white (*Hagiomantis* sp.) praying mantis morphospecies against greenish-brown and whitish trunk backgrounds in the southern Amazon rainforest. The big circles indicate mean values while the whiskers represent upper and lower confidence intervals (CI 95%) estimated from the mixed-effects model (see details in the main text). Small circles represent raw data.

The time that human predators spent to find the artificial mantis models and the distance at which they spotted them differed between model types, but the direction of the effect depended on the trunk background (Table 2). The time to find green models against greenish-brown trunks was more than five times longer than to find white models against the same trunks. Similarly, the time that humans spent to find white models against whitish trunks was more than three times longer than to find green models against similar trunks (Fig. 5). An opposite pattern was observed for the encounter distance, at which more camouflaged models (i.e., green models against greenish-brown trunks and white models against whitish trunks) required shorter distances to be found by humans compared to those more conspicuous (Fig. 5).

**Table 2:** Predation experiment using humans as predators searching for mantis models against trunk backgrounds in the southern Amazon rainforest. Summary results of the analysis of variance applied to linear mixed-effects models testing differences in the searching time (in seconds) and the encounter distance (in meters) of human predators “hunting” realistic praying mantis paper models resembling white (*Hagiomantis* sp.) and green (*Liturgusa* sp.) morphospecies against different trunk backgrounds (greenish-brown and whitish trunks). For both models, predator and tree identity were included as random factors to control for data dependence. Significant differences are shown in bold.

		Searching time (s)			Encounter distance (m)		
	*df*_num_ / *df*_den_	*MS*	*F*	*p*	*MS*	*F*	*p*
Background	1/4	0.016	0.16	0.713	7.413	3.11	0.152
Mantis model	1/224	0.363	3.49	0.063	14.829	6.23	**0.013**
Background * mantis model	1/224	24.896	239.47	** < 0.001**	747.272	313.84	** < 0.001**

## Discussion

In this study, we integrate the use of colour analysis and experiment in the field to assess how bark praying mantises of different morphospecies may employ distinct camouflage strategies to occupy variable backgrounds in the Amazon rainforest. Our results strongly suggest local and microhabitat adaptations between praying mantis body colour patterns and their trunk background. Ultimately, the combination between increased colour matching and disruptive coloration promotes efficient camouflage for praying mantises, as it increases search time and decreases encounter distances by potential predators.

As predicted, the different praying mantis morphospecies vary in their level of background matching and disruptive coloration among background types, with better adjustments favouring, in the major scenarios, at occupied trunk and microhabitat scale adaptations. It is important to point out that most tree trunks in the Amazon rainforest are highly heterogeneous regarding their colour pattern (Fig. 1), with bryophyte and lichen patches randomly distributed over trunks, resulting in colour and pattern changes within a few centimetres. Even though praying mantises are very fast, frequently shifting from the bottom to the top of the tree in a few seconds (Authors, *personal observations*), the individuals of the three morphospecies we study here still exhibit very low mean JND values and high GabRat values against fixed points in trunk backgrounds. However, in the case of green praying mantis (*Liturgusa* sp.), for example, the lack of a representative microhabitat patch (i.e., bryophytes in greenish-brown trunks) may lead to a poor local background matching. Moreover, the mean chromatic contrast of all mantis morphospecies against the occupied trunks regardless of the scale was broadly < 3.00 JND, indicating that avian predators will have difficulty to detect praying mantis against their trunks under natural light conditions^48^.

The trunk-dwelling lifestyle of these animals may also reinforce that natural selection would favour highly efficient luminance matching against potential predators in Liturgusidae praying mantis. However, under medium and long distances, the praying mantises of all morphospecies would be easily detectable from potential predators, as the mean luminance value for all combinations are consistently larger than 3 units^48^. Luminance contrasts are though very variable, varying from low (~ 3 JND) to very high (> 30 JND units) between trunk conditions and morphospecies, which indicates a high heterogeneity in the brightness of trunk backgrounds. Such variable luminance contrasts could also indicate a generalist background matching, in which the praying mantis matches in the luminance to some degree with several backgrounds but not perfectly with any of them, a type of compromise luminance^49^. However, a recent study demonstrated that, unlike colour diversity, background luminance diversity did not prevent the detection of artificial prey by avian predators^50^, suggesting that background colour diversity may be more important to some environments as the bark praying mantises in the Amazon rainforest.

Only a few numbers of studies have quantified the role of microhabitat behavioural selection as a strategy to improve individual camouflage^3,51–53^. In order to be successful and increase prey survival, camouflage may benefit from prey habitat choices towards matching backgrounds within broad or fine scales^4,8^. For example, artificial models of lichen moths *Declana atronivea* presented higher survival rates against avian predators when models were fixed on specific positions of tree trunks composed by lichens but had lower survival on bark lichen-free substrates^54^. These results can be related to our study system, as green praying mantis (*Liturgusa* sp.) exhibited improved background matching in terms of both colour and luminance contrasts against bryophyte patches (microhabitat scale). However, differently from moths, praying mantises are predators and very mobile animals, so the challenge to match specific positions of trunks is even higher, as they use trunks not only to rest but also for foraging^55^. Considering that bryophyte patches are randomly distributed over trunks and green praying mantises exhibited a high match to this microhabitat, it is also possible that individuals of this species could benefit from a masquerade camouflage strategy^56^. When occupying portions of the trunks without bryophyte cover, green mantis could be detected by predators but being recognized as a small patch of bryophyte, especially when viewed in a flat position and at long distances by a generalist avian predator^57^. New studies may indicate whether praying mantises can optimize their colour matching by selecting microhabitat patches and orient the body to specific positions to improve concealment or if individuals would remain immobile close to those patches, which would make them similar to bryophyte or lichen patches and favour a masquerade strategy.

Disruptive coloration is one of the most widespread camouflage strategies in nature and has been studied for a long time^58,59^. However, only recently, new tools allowed us to quantify animal disruption and how this strategy is affected by different backgrounds, but with seldom examples across taxa^16,60–62^. In addition, several studies show that disruptive coloration can operate simultaneously or independently to background matching, as the high contrast of markings especially in the body edges promotes advantages less dependent on the background similarity^59,63, 64^. Our results suggest that disruptive coloration can favour green mantises (*Liturgusa* sp.) on a local scale when the level of background matching is poor. Similar outcomes were observed for juvenile shore crabs (*Carcinus maenas*), in which camouflage has seen to be improved by increasing edge disruption when background matching was not highly effective^41^. Similarly, white (*Hagiomantis* sp.) and grey praying mantises (Liturgusidae) presented intermediated degrees of edge disruption against both whitish and greenish-brown trunks, which reinforces the less dependence of this strategy on substrate types and its high efficiency against detection in heterogeneous backgrounds^57^.

Field predation experiments have been considered as important tools to determine the protective role of animal coloration in natural settings^21,58^. These experiments are important because they allow researchers to change, add or subtract a given colour trait and test it in real or simulated situations^65,66^. Here, we used experimental models to test the camouflage efficiency of realistic praying-mantis models against natural backgrounds in the Amazonian rainforest for the first time. Differently from recent studies^37,54^, we purposely did not insert the models directly into lichen or bryophyte patches but in random locations on the trunk. This decision was based on our observations, which indicated that mantises usually remain in the same trunk areas, making small positional changes, and significantly altering their position only when disturbed or when they are hunting for prey. Therefore, in our experiment we show that from a local tree-type scale, the camouflage of green praying mantises (*Liturgusa* sp.) against greenish-brown trunks is efficient to reduce predation risks, even though the visual models indicated better matching to bryophyte microhabitat. In addition, white praying mantises (*Hagiomantis* sp.) also presented higher survival against whitish trunks, which matches the visual model contrasts. Since the models we created sought to mimic the silhouette and the colour patterns of real praying mantises as much as possible, some level of disruptive coloration was maintained during the experiment and cannot be dissociated from our results. However, it is important to note that we used humans as predators because several other studies have provided evidence about the similarity in predation response to artificial targets between human and avian predators, which allow us to discuss the evolutionary forces shaping animal coloration in nature^21,22^. Other predators, such as invertebrates, possess different visual systems and may differ in predatory patterns^67^. Therefore, our field experiment is one of the few studies to date testing how different morphospecies can use different concealment strategies (i.e., background matching over local and microhabitat scales and disruptive coloration) to evade detection in heterogeneous environments such as the Amazon rainforest.

Although Liturgusidae praying mantises are known to be highly dependent on camouflage due to their trunk–dwelling lifestyle, to our knowledge, no study has objectively quantified the degree of camouflage of these important predators considering their natural backgrounds^17^. We reinforce the importance of molecular tools (e.g., DNA Barcoding) to minimize research bias and identification mistakes, especially in juvenile individuals and in studies with unexplored taxonomic groups. Our study shows that different morphospecies, despite the similarity in morphology and behaviour, can use different camouflage strategies that are highly effective on a tree trunk and microhabitat scale to avoid their predators. The Liturgusidae family contains 19 genera of praying mantises around the world, with all the described species being highly dependent on tree trunks to forage and avoid predation^68^. Therefore, the increased camouflage effectiveness of these praying mantises associated to their tree-dwelling lifestyle suggests a high irradiation process for the occupation of different forest habitats mediated by natural selection, which provides a new future research area^18^. Phylogenetic reconstruction could be an important tool to understand how camouflage patterns evolved and which ecological processes shaped the distribution of praying mantises in highly heterogeneous landscapes^69^.

In conclusion, we bring new evidence of the use of different camouflage strategies by praying mantis of different species that are virtually unknown in the Amazon rainforest. The high diversity and heterogeneity of colour patterns of tropical rainforests habitats can select for a diversity of animal adaptive responses, ranging from optimal camouflage to general background and microhabitat scales. Furthermore, our study opens a research avenue for new studies testing hypotheses on local adaptation in highly heterogeneous environments, such as Amazonian trees, as well as potential adaptations for active habitat choice and ontogenetic or substrate-dependent colour change in praying mantises^46,70^. Our study is one of the few to integrate field predation experiment with realistic prey based on vision models, to compare background matching and disruptive coloration camouflage strategies and to access the functional level of such strategies against different backgrounds, which ultimately presents an ideal system for further investigation about the adaptive value of camouflage strategies under sympatric conditions. Furthermore, our study reveals a wide and underexplored field of research about the evolutionary and ecologic processes shaping camouflage diversification in natural systems, widening the knowledge on the diversity of cryptic species so far unknown at unexplored ecosystems.

## Data and code availability

All data generated and analysed for the current study, as well as the R codes, are available on Figshare (https://figshare.com/s/dcbd05dcd2f9d7480124).

### Supplementary Information

Supplementary Information.
