# Author Correction: Crypsis by background matching and disruptive coloration as drivers of substrate occupation in sympatric Amazonian bark praying mantises

**DOI:** 10.1038/s41598-023-49317-5

**Published:** 2023-12-15

**Authors:** João Vitor de Alcantara Viana, Rafael Campos Duarte, Camila Vieira, Pablo Augusto Poleto Antiqueira, Andressa Bach, Gabriel de Mello, Lorhaine Silva, Camila Rabelo Oliveira Leal, Gustavo Quevedo Romero

**Affiliations:** 1 Programa de Pós-Graduação em Ecologia, Instituto de Biologia, Universidade Estadual de Campinas, Campinas, São Paulo Brazil; 2 Laboratório de Interações Multitróficas e Biodiversidade, Departamento de Biologia Animal, Instituto de Biologia, Universidade Estadual de Campinas (UNICAMP), CP 6109, Campinas, São Paulo CEP 13083-970 Brazil; 3 Universidade Federal Do ABC, São Bernardo Do Campo, São Paulo CEP 09606-045 Brazil; 4 Centre for Ecology and Conservation, College of Life and Environmental Sciences, University of Exeter, Penryn Campus, Penryn, TR10 9FE UK; 5 Departamento de Ciências Básicas, Universidade de São Paulo (USP), Campus de Pirassununga, Pirassununga, São Paulo CEP 13635-900 Brazil; 6 Programa de Pós-Graduação Em Ecologia E Conservação da Biodiversidade, Instituto de Biociências, Universidade Federal de Mato Grosso, Avenida Fernando Corrêa da Costa, N° 2367, Boa Esperança, Cuiabá 78060900 Brazil

Correction to: *Scientific Reports* 10.1038/s41598-023-46204-x, published online 15 November 2023

In the original version of this Article, a reference was omitted.

Reference 42:

‘van den Berg, CP, Troscianko, J, Endler, JA, Marshall, NJ, Cheney, KL. Quantitative Colour Pattern Analysis (QCPA): A comprehensive framework for the analysis of color patterns in nature. Methods Ecol Evol. 2020; 11: 316–332. 10.1111/2041-210X.13328’.

As a result, in the Materials and Methods section,

“We also used blue tit visual model, reducing our channels to the double cones, and set an acuity value of 6 cycles per degree at 1 m distance because these values closely resemble the acuity for other small avian predators that forage on trunks20^,^39^,^41.”

now reads,

“We also used blue tit visual model, reducing our channels to the double cones, and set an acuity value of 6 cycles per degree at 1 m distance because these values closely resemble the acuity for other small avian predators that forage on trunks20^,^39^,^41,42.”

The References have been renumbered.

The original Article has been corrected.

