# Supplementary material for: Crypsis by background matching and disruptive coloration as drivers of substrate occupation in sympatric Amazonian bark praying mantises
Source: Sci Rep. 2023 Nov 15;13:19985. doi: 10.1038/s41598-023-46204-x (PMC10652001; doi:10.1038/s41598-023-46204-x)
Supplement: Supplementary file 1 — Supplementary Information. [file 41598_2023_46204_MOESM1_ESM.docx]

**Supplementary material 1.**

**Crypsis by background matching and disruptive coloration as drivers of substrate occupation in sympatric Amazonian Bark praying mantises**

**Supplementary tables.**

**Table S1.** Individuals of praying mantis sampled in the Amazonian Rainforest included in cytochrome c oxidase I (COI) Barcode analysis. Annealing temperature for PCR amplification and GenBank accession number are provided.

| **Morphotype Specimen** | **Colour morph** | **Annealing temperature (°C)** | **GenBank acession number** |
| --- | --- | --- | --- |
| *Hagiomantis* sp. | White | 50 | OR073655 |
| *Hagiomantis* sp. |  | 50 | OR073656 |
| *Hagiomantis* sp. |  | 50 | OR073657 |
| *Hagiomantis* sp. |  | 45 | OR073658 |
| *Hagiomantis* sp. |  | 45 | OR073659 |
| *Liturgusa* sp. | Green | 50 | OR073660 |
| *Liturgusa* sp. |  | 50 | OR073661 |
| *Liturgusa* sp. |  | 50 | OR073662 |
| *Liturgusa* sp. |  | 45 | OR073663 |
| Liturgusidae sp. | Gray | 45 | OR073664 |
| Liturgusidae sp. |  | 54 | OR073665 |
| Liturgusidae sp. |  | 45 | OR073666 |
| Liturgusidae sp. |  | 54 | OR073667 |
| Liturgusidae sp. |  | 45 | OR073668 |

**Table S2.** Pairwise Kimura two-parameter distance of praying mantis considering 640 bp cytochrome c oxidase I (COI) sequences. Values in bold are showing distance higher than 3%, indicating that individuals with different colour do not belong to the same species (Hebert et. al 2003).

|  | 1 | 2 | 3 | 4 | 5 | 6 | 7 | 8 | 9 | 10 | 11 | 12 | 13 |
| --- | --- | --- | --- | --- | --- | --- | --- | --- | --- | --- | --- | --- | --- |
| 1 - JO02_"white" | - |  |  |  |  |  |  |  |  |  |  |  |  |
| 2 - JO06_"white" | 0.002 | - |  |  |  |  |  |  |  |  |  |  |  |
| 3 - JO11_"white" | 0.002 | 0.000 | - |  |  |  |  |  |  |  |  |  |  |
| 4 - JO26_"white" | 0.009 | 0.007 | 0.007 | - |  |  |  |  |  |  |  |  |  |
| 5 - JO27_"white" | 0.004 | 0.002 | 0.002 | 0.009 | - |  |  |  |  |  |  |  |  |
| 6 - JO30_"white" | 0.009 | 0.007 | 0.007 | 0.007 | 0.009 | - |  |  |  |  |  |  |  |
| 7 - JO31_"white" | 0.007 | 0.005 | 0.005 | 0.009 | 0.005 | 0.009 | - |  |  |  |  |  |  |
| 8 - JO14_"green" | **0.181** | **0.179** | **0.179** | **0.174** | **0.181** | **0.179** | **0.181** | - |  |  |  |  |  |
| 9 - JO21_"green" | **0.181** | **0.179** | **0.179** | **0.174** | **0.181** | **0.179** | **0.181** | 0.000 | - |  |  |  |  |
| 10 - JO22_"green" | **0.181** | **0.179** | **0.179** | **0.174** | **0.181** | **0.179** | **0.181** | 0.000 | 0.000 | - |  |  |  |
| 11 - JO29_"green" | **0.179** | **0.177** | **0.177** | **0.172** | **0.179** | **0.177** | **0.179** | 0.004 | 0.004 | 0.004 | - |  |  |
| 12 - JO32_"grey" | **0.185** | **0.183** | **0.183** | **0.178** | **0.185** | **0.183** | **0.185** | **0.142** | **0.142** | **0.142** | **0.142** | - |  |
| 13 - JO33_"grey" | **0.187** | **0.185** | **0.185** | **0.180** | **0.183** | **0.185** | **0.181** | **0.148** | **0.148** | **0.148** | **0.148** | 0.014 | - |

**Supplementary Figure 1.**


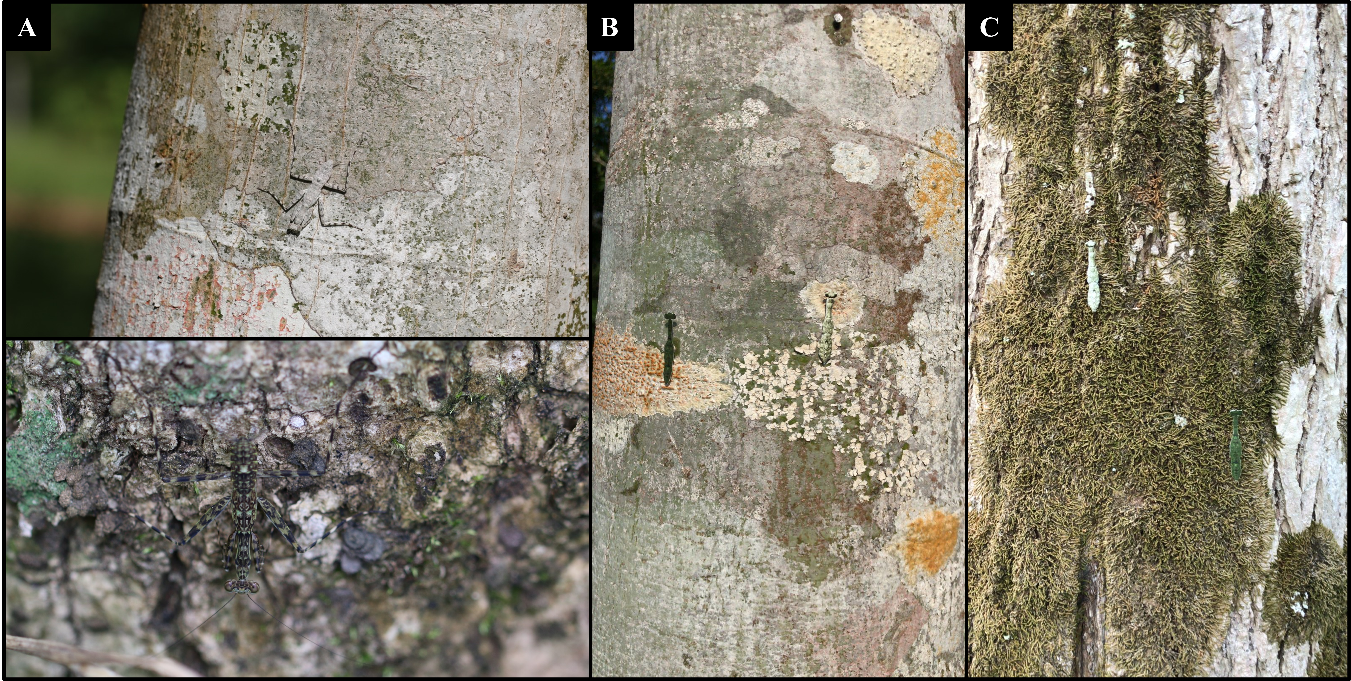


**Figure S1**. Field predation experiment. The photos depict the field predation experiment with human predators in the southern Amazon rainforest. Picture A) shows in the upper panel the white morphospecies (*Hagiomantis* sp.) resting on whitish trunks and in the below panel, the green morphospecies (*Liturgusa* sp.) resting on greenish-brown trunks. Panel B shows the pair models of paper mantis (white and green) placed on whitish trunks covered by lichens. Panel B shows the pair models of paper mantis (white and green) placed on greenish-brown trunks covered by random patches of bryophytes. In the experiment, the pair of paper model mantis were randomly placed on both trunk types.
